# Modulating activity of PVN neurons prevents atrial fibrillation induced circulation dysfunction by electroacupuncture at BL15

**DOI:** 10.1186/s13020-023-00841-6

**Published:** 2023-10-17

**Authors:** Jingya Wang, Qiumei Zhang, Lin Yao, Teng He, Xinyi Chen, Yang Su, Shengxuan Sun, Mengyue Fan, Jinglan Yan, Taiyi Wang, Meng Zhang, Feng Guo, Shiqing Mo, Manqi Lu, Meixia Zou, Liangjie Li, Qing Yuan, Huashan Pan, Yongjun Chen

**Affiliations:** 1 Institute of Acupuncture and Moxibustion, Shandong University of Traditional Chinese Medicine, Jinan, 250355 People’s Republic of China; 2 South China Research Center for Acupuncture and Moxibustion, Guangzhou University of Chinese Medicine, Guangzhou, 510006 People’s Republic of China; 3 Institute of Physical and Health, Guangzhou University of Chinese Medicine, Guangzhou, 510006 People’s Republic of China; 4 Guangdong Chaozhou Health Vocational College, Chaozhou, 521000 People’s Republic of China; 5 School of Pharmaceutical Sciences, Guangzhou University of Chinese Medicine, Guangzhou, 510006 People’s Republic of China

**Keywords:** Circulation dysfunction, Atrial fibrillation, Electroacupuncture, Sympathetic activity, Paraventricular nucleus of hypothalamus

## Abstract

**Background:**

Circulation dysfunction is a major contributing factor to thrombosis in patients with atrial fibrillation (AF) for which effective interventions are lacking. Growing evidence indicates that regulating the paraventricular nucleus (PVN), an autonomic control center, could offer a novel strategy for treating cardiovascular and circulatory diseases. Concurrently, electroacupuncture (EA) at Xinshu (BL15), a form of peripheral nerve stimulation, has shown efficacy in treating several cardiovascular conditions, although its specific mechanism remains unclear. This study aimed to assess the impact of EA at BL15 on circulatory dysfunction in a rat AF model and investigate the pivotal role of PVN neuronal activity.

**Methods:**

To mimic the onset of AF, male SD rats received tail intravenous injection of ACh-CaCl_2_ and were then subjected to EA at BL15, sham EA, or EA at Shenshu (BL23). Macro- and micro-circulation function were evaluated using in vivo ultrasound imaging and laser doppler testing, respectively. Vasomotricity was assessed by measuring dimension changes during vascular relaxation and contraction. Vascular endothelial function was measured using myograph, and the activation of the autonomic nerve system was evaluated through nerve activity signals. Additionally, chemogenetic manipulation was used to block PVN neuronal activation to further elucidate the role of PVN activation in the prevention of AF-induced blood circulation dysfunction through EA treatment.

**Results:**

Our data demonstrate that EA at BL15, but not BL23 or sham EA, effectively prevented AF-induced macro- and micro-circulation dysfunction. Furthermore, EA at BL15 restored AF-induced vasomotricity impairment. Additionally, EA treatment prevented abnormal activation of the autonomic nerve system induced by AF, although it did not address vascular endothelial dysfunction. Importantly, excessive activation of PVN neurons negated the protective effects of EA treatment on AF-induced circulation dysfunction in rats.

**Conclusion:**

These results indicate that EA treatment at BL15 modulates PVN neuronal activity and provides protection against AF-induced circulatory dysfunction.

**Supplementary Information:**

The online version contains supplementary material available at 10.1186/s13020-023-00841-6.

## Introduction

Circulation dysfunction, encompassing hemodynamic changes, vascular injury, and hypercoagulability, constitutes a common pathological feature observed in atrial fibrillation (AF) patients during clinical trials [1, 2]. Research has highlighted that both blood constituents and vascular factors play crucial roles in abnormal blood flow, leading to thrombosis in AF patients, apart from left atrial appendage contractile dysfunction [3, 4]. Notably, AF patients exhibit activation of the coagulation cascade and the formation of pathological thrombi in blood vessels [5–7]. Additionally, elevated levels of vascular endothelial damage markers have been observed in various types of AF [8, 9]. Current approaches for preventing embolism in AF individuals involve anticoagulation therapy and percutaneous left atrial appendage contractile closure [10]. However, these treatments come with bleeding risks, such as life-threatening gastrointestinal and intracranial hemorrhage [11]. Moreover, the invasive nature of left atrial appendage contractile closure may lead to blood clot formation around the device and pericardial effusion [12, 13]. Therefore, there is an urgent need for a safe and effective treatment to mitigate AF-induced circulation dysfunction.

The paraventricular nucleus (PVN) is a critical autonomic control center in the brain that plays a role in regulating cardiovascular function by modulating sympathetic vasomotor tone [14], autonomic stress responses, and various neuroendocrine factors [15]. It achieves this by receiving and integrating peripheral sympathetic activity and transmitting integrated information through relevant neural signaling pathways [16]. Extensive research has demonstrated that the autonomic nervous system significantly influences blood volume regulation [17], blood pressure[18], cerebral artery occlusion [19], cardiovascular responses to stress [20], and various cardiovascular diseases and circulatory injuries [17], such as hypertension [21], arrhythmia [22], and chronic heart failure [23]. Our previous study reported increased PVN neuron activity in AF rats [24]. This mounting evidence supports the potential of regulating higher central regions, particularly the PVN, as a novel therapeutic approach for cardiovascular and circulatory diseases. Nonetheless, additional experimental evidence is needed to ascertain whether the PVN can be a key target for treating circulatory dysfunction in atrial fibrillation.

Electroacupuncture (EA), a form of peripheral nerve stimulation used to improve organ function, has emerged as a promising treatment option for complex and refractory conditions, including hypertension, heart failure, and others [25–28]. In traditional Chinese medicine theory, ‘‘back-Shu’’ points are commonly utilized to address disorders of their corresponding ‘‘visceral organs’’ [29]. Prior investigations have demonstrated that acupuncture or EA stimulation [30–33] at Xinshu (BL15) effectively treats various cardiovascular ailments, such as coronary heart disease, hypertension, and myocardial infarction angina pectoris, among others [34–36]. These studies have indicated that BL15 stimulation may enhance microcirculation under the aforementioned pathological conditions, but the underlying mechanism remains unclear. Previous findings from our research have shown that EA can modulate autonomic nerve balance, which is linked to the regulation of several brain regions, including the PVN [24]. Moreover, EA treatment has been shown to regulate the discharge frequency of PVN neurons in a rat model of myocardial ischemia [37]. However, it remains unclear whether EA at BL15 can ameliorate circulatory dysfunction in atrial fibrillation and the underlying mechanisms involved. Thus, the present study aimed to evaluate the therapeutic effect of EA at BL15 on circulatory dysfunction in a rat AF model and explore the pivotal role of PVN neuron activity.

## Materials and methods

### Experimental animals

Male Sprague–Dawley rats weighing 300–350 g were procured from the Laboratory Animal Center at Guangzhou University of Chinese Medicine (GZUCM). The rats were housed under controlled light cycles (12 h of light, 12 h of dark) and maintained at a temperature of 23 ± 2 °C. Standard rat chow and water were provided ad libitum. Animal care and experimental protocols were approved by the Institutional Animal Care and Use Committee of GZUCM (Protocol Number: 20201124005).

### Administration of acetylcholine (ACh) and CaCl_2_

The AF animal model was induced by the intravenous injection of Acetylcholine (ACh) and CaCl_2_, as previously described [24]. Briefly, CaCl_2_ (Sigma, Saint Louis, USA) was dissolved in saline, and ACh (Sigma, Saint Louis, USA) was added under protection from light. The AF rat model was established by daily intravenous injection of 1 mL/kg of the mixture (ACh, 66 μg/mL, and CaCl_2_, 10 mg/mL) for 10 consecutive days. Prior to injection, rats were anesthetized with 2% isoflurane and secured on an animal heating pad with adhesive tape on their abdomen.

### EA treatment

Rats in the electroacupuncture (EA) group underwent EA treatment at the left ‘‘Xinshu’’ (BL15, 5 mm lateral to the spine at T5 level) or ‘‘Shenshu’’ (BL23, located 5 mm lateral to the posterior midline, on the level of the lower border of the spinous process of the 2nd lumbar vertebra) [38]. EA treatment was performed for 20 min per day for 10 consecutive days at 2 mA and 2 Hz, starting 10 min after ACh-CaCl_2_ administration, as previously described [39]. To account for potential effects of anesthesia or body fixation, rats in all groups were anesthetized with isoflurane (2%) and secured on the animal heating pad using adhesive tape in a standardize position on their abdomen. One needle (0.25 mm × 13 mm, Beijing Hanyi Medical Instruments Co Ltd, Beijing, China) was inserted at a 45-degree angle to a depth of 4–5 mm into the left BL15 or ‘‘Shenshu’’ (BL23), with the other needle inserted 5 mm away. The two needles were connected to the positive and negative poles of a stimulator (Master-8, AMPI, Israel), respectively. For the sham EA treatment, stainless steel needles were inserted 2–3 mm into the same points as the EA group, but without electrical current injection.

### Preparation of isolated hearts

All rat hearts were isolated and perfused with the Langendorff system (Model: SGL, scope research institute of electrophysiology, China) as previously described [40]^.^ After euthanization with an intraperitoneal injection of 2% pentobarbital sodium (0.3 ml/100g), the chest was opened, and the hearts were rapidly excised. The hearts were perfused with oxygenated (95% O_2_, 5% CO_2_) Tyrode's solution through the aorta until they beat rhythmically. Subsequently, the blood was flushed out, and the hearts were placed in the Langendorff system under continuous perfusion with oxygenated Tyrode's solution at 38 °C. The Tyrode's solution composition consisted of 134 mM NaCl, 4.5 mM KCl, 0.5 mM MgCl_2_, 2 mM NaH_2_PO_4_, 23 mM NaHCO_3_, 1.8 mM CaCl_2_, and 5.5 mM glucose, equilibrated with 95% O_2_ and 5% CO_2_ to maintain a pH of 7.4.

### AF inducibility and ERP analysis

The electrocardiogram (ECG) signal from the isolated heart was recorded using an electrophysiological mapping system (Mapping Lab, EMS64-USB-1003, UK) [41]. In brief, a stimulation electrode was inserted into the left atrium of the heart, while two ECG recording electrodes were placed in the right atrium and left ventricle, respectively. The pacing current threshold was determined by a train stimulation, increasing the intensity from 1 to 3 mA with a duration of 2 ms. The minimum current required for successful pacing of the heart was considered the systole threshold for current intensity. The experimental stimulus current was then set at 2 times the systole threshold current amplitude. To assess AF inducibility, S1−S1 stimulations were applied with two stimulation steps at the frequency of 50 Hz. The same stimulation was repeated eight times to calculate AF inducibility and duration. To determine the effective refractory period (ERP) in all groups, a trained stimulation 1 (S1, 5 Hz) was repeated ten times to pace the heartbeat, which was considered to be the basic stimulation. The interval between S1−S1 was set to 100 ms. Stepwise decreasing electrical stimulation 2 (S2, 5 Hz) was applied, repeating S1−S2 ten times, with the interval of S1−S2 set to 60 ms and a reduction of 5 ms per cycle until S2 failed to induce a complete heartbeat signal trace. The last S1−S2 interval time was recorded as the ERP.

### In vivo ultrasound imaging and assessment

Vascular wall and hemodynamic function in the carotid and femoral arteries were studied using a Vevo 2100 Ultrasound Image system (Fujifilm Visualsonics, Canada), with an ultrasonic coverage of 22–55 MHz and a central frequency of 40 MHz [42]. Measurements were randomly obtained and labeled by one operator. Rats were anesthetized with 4% isoflurane in a chamber with 1 L/min of medical oxygen for 3–5 min, after which the isoflurane concentration was adjusted to 2% to maintain anesthesia during measurements. Hair on the legs and cervix of rats was removed using depilatory cream in the supine position. The probe was placed parallel to the rat's neck to locate the left and right carotid arteries. M-mode images were used to measure the diameters of the right and left common carotid arteries during relaxation and contraction, as well as the mean blood velocity (MV) of these arteries. Blood flow and diameter differences during relaxation and contraction were calculated as follows: blood flow (mL/min) = MV × π (vessel diameter/2)^2^; diameter difference (mm) = maximal diameter during vessel relaxation—minimum diameter during vessel contraction. Next, the probe was moved to the leg bone to locate the femoral artery and obtain measurements, including the diameters of the right and left femoral arteries during relaxation and contraction, and the MV of these arteries. The blood flow and diameter differences during relaxation and contraction of the femoral arteries were calculated using the same formulas as above.

### Laser Doppler measurement

In vivo microcirculation of the lower limbs and kidneys was assessed using Laser Doppler flowmetry (PERIMED PeriFlux5000 system, Sweden), as previously described [43]. The rats were anesthetized with 2% isoflurane and placed on a heating pad at 37 °C to maintain their body temperature. The plantar surfaces of the lower limbs were positioned on a dark surgical towel after gentle removal of hair using hair removal cream. Tissue perfusions were quantified in regions of interest (ROI) within the upper 200–300 μm of the skin, and the mean Perfusion Unit (PU) was recorded. The same procedure was applied for PU assessment in the kidneys. The rats were placed in a dissecting pan with their ventral side exposed, and a cut was made from the middle of the body to the hindlimbs to expose the kidneys. The kidney surface was gently moistened with saline during the experiment. The laser scan head was adjusted to achieve the best image quality, and a constant distance was maintained for all measurements.

### Enzyme-linked immunosorbent assay (ELISA)

Following the administration of the ACh and CaCl_2_ mixture and the 10 day EA treatment, all rats were anesthetized with an intraperitoneal injection of 2% pentobarbital sodium (0.3 mL/100 g) and dissected after the disappearance of the foot withdrawal reflex. Blood samples (3 ml) were collected from each rat through intracardiac puncture. D-Dimer and fibrinogen (FG) levels were analyzed by ELISA (Elabscience Biotechnology Co., Ltd. Shanghai, China) according to the manufacturer's instructions. Blood samples were transferred into empty tubes and centrifuged at 3000 g for 10 min. The liquid supernatant serum was collected and stored at − 80 °C for subsequent use.

### Myograph

The carotid/femoral arteries were removed and placed in a 4 °C chilled Krebs solution (NaCl, 118 mM; sodium bicarbonate, 25 mM; glucose, 5.6 mM; potassium chloride, 4.7 mM; KH_2_PO_4_, 1.2 mM; MgSO_4_ 7H_2_O, 1.17 mM; and CaCl_2_·2H_2_O, 2.5 mM). The residual blood and perivascular fat were carefully removed before the myograph experiments. Vascular endothelial function was evaluated using a myograph (No: DMT620, Danish Myo Technology, Aarhus, Denmark), as previously described [44]. The isometric force was recorded using a PowerLab/8SP data acquisition system (Software Chart, Version 5, AD Instrument, Colorado Springs, CO, USA). The isolated carotid/femoral arteries were cut into 2 mm rings and placed in 5 mL baths of myograph, equilibrated for 1 h in Krebs solution (37 °C) under a resting tension of 5.0 mN, and bubbled with 95% O_2_ and 5% CO_2_. Subsequently, phenylephrine (PE: 10–6 M, code: P1250000, Sigma, Saint Louis, USA) was used to precontract the vascular rings. Once the vessel contraction reached a plateau, acetylcholine (ACh: 10–9 to 10–5 M, endothelium-dependent vasodilator, No: A2661, Sigma, Saint Louis, USA) or the NO donor sodium nitroprusside (SNP: 10–9 to 10–5 M, endothelium-independent vasodilator, No: 71778, Sigma, Saint Louis, USA) was added to the chamber to produce a cumulative concentration–response vasorelaxation curve.

### Electrocardiogram In vivo

The electrocardiogram (ECG) was conducted following the procedures described previously [45]. Briefly, the last injection of the mixed solution (ACh and CaCl_2_) was administered through the caudal veins of the rats. After a 5 min interval, the rats were anesthetized with 2% isoflurane and positioned in a supine position on an animal heating pad set at 37 ℃. Needle-like electrodes of a multichannel physiological recorder (PowerLab 16/35, AD Instruments Pty, Australia) were placed on both the right upper limb and one left lower limb (standard II lead). The rats were recorded once their respiration stabilized, and their heart rate reached 350 ± 25 beats/min. The ECG signals (5 mV, 1 k/s sampling rate) were analyzed using LabChart 8.0 software (AD Instruments Pty, Australia). Heart rate variability (HRV) analysis was conducted following established protocols [46, 47]. For frequency-domain analysis, the absolute power of different frequency bands commonly used in rats, including low frequency (LF) and high frequency (HF), was determined. The HF (0.75–2.5 Hz) peak is generally indicative of cardiac vagus nerve activity, while the LF (0.2–0.75 Hz) is often associated with a dominant sympathetic component.

### Autonomic nerve activity recording

The cardiac sympathetic and vagus nerve activity were recorded following established procedures [48]. In brief, the rats were placed in a supine position on a warm heating pad and anesthetized with 4% isoflurane for induction and 2% for maintenance. An incision was made at the neck midline to expose the left carotid artery, and the trachea was separated with a glass needle. This allowed access to the cervical sympathetic nerve medial to the carotid and the vagus nerve lateral to the carotid. The epineurium was carefully peeled after fixing the sympathetic and vagus nerves with a glass needle. A platinum-iridium alloy wire cuff electrode (Kedou Brain Computer Technology, China) was used to hook onto the cardiac sympathetic or cervical vagus nerve, with a referred electrode inserted subcutaneously into the ipsilateral neck. The signal of nerve activity was recorded using a recording system (Bio-Signal Technologies, USA) and analyzed with Neuroexplorer 5.0 software (Plexon, USA). The signal was band passed at 100–1000 Hz.

### Virus constructs and chemogenomic manipulation

For the chemogenetic manipulation experiments of paraventricular nucleus of the hypothalamus (PVN) neurons, rats were anesthetized with 2% isoflurane and placed in a surgical stereotactic apparatus (RWD Instruments, China). The skull was leveled using bregma and lambda landmarks, and injections were made into the PVN at an angle of 6°: ± 1.2 mm from the bregma, 2.05–2.25 mm lateral from the midline, and 7.95 mm ventral to the skull. Adeno-associated virus (AAV) expressing hM3D (Gq) (pAAV-SYN-HA-hM3D (Gq)2A-mCherry-3FLAG, Viral titer: 1.22E + 13) obtained from Obio Technology, Shanghai, was used. Bilateral viral injections of 100 nL virus were administered with two 1 μL Hamilton Syringes connected to a micro pump (RWD Instrument, China). The rat body temperature was maintained with a heating pad set at 36℃. To allow for viral expression in the PVN, clozapine N-oxide (CNO, APExBIO) intraperitoneal injection was performed 60 min before EA treatment at 3 weeks after the initial viral injection. Artery imaging baseline was recorded before CNO administration and after virus injection. Rats were injected with 1 mg/kg CNO for hM3Dq activation, while control rats were injected with the same amount of vehicle. Experimenters were blinded to viral identity at the time of viral injection and to rat identity until after the experiments were completed.

### Immunofluorescence

Rats were transcardially perfused with 0.9% NaCl followed by 4% paraformaldehyde in 0.1 M PB (pH 7.4; 4 °C) after deep anesthesia with an intraperitoneal injection of 2% pentobarbital sodium (0.3 ml/100 g). The brains were immediately removed and postfixed with 4% paraformaldehyde overnight. The tissues were then dehydrated in 15% and 30% sucrose in 0.1 M PBS at 4 °C for 48 h. OCT-embedded blocks were sectioned to a thickness of 40 μm. Sections from each group were rinsed in 0.01 M PBS three times and blocked for 2 h with blocking liquid (5% goat serum and 0.2% Triton-100 in 0.01 M PBS) at room temperature. The sections were then incubated with rabbit anti-c-Fos antibody (1:1000, mAb#2250, CST) in blocking buffer overnight at 4 °C. The following day, the free-floating sections were washed with 0.01 M PBS three times and incubated with the secondary antibody Alexa Fluor 488 goat anti-rabbit (1:500; ab150077, Abcam) at room temperature for 2 h. DAPI was applied for nucleus staining for 5 min. After being washed in 0.01 M PBS, the brain slices were cover-slipped. Images were captured using a microscope (Axio Imager.A2, ZEISS, Germany), and analysis was performed using NIH Image J software (Bethesda, MD, USA).

### Statistical analyses

Statistical analyses were conducted using SPSS 21.0 (Chicago, IL, USA) and GraphPad Prism version 5.0 (San Diego, CA, USA). All data were expressed as mean ± SEM. Statistical differences were determined using analysis of variance (one-way or two-way ANOVA), and Bonferroni multiple comparison test was used for paired comparisons between groups when the data met a normal distribution. For data that did not meet a normal distribution, Dennett’s tests were employed for pairwise comparison between groups. Experimental values of relaxation were calculated relative to the maximal changes in the contraction produced by PE, respectively, taken as 100% in each tissue. Curves were fitted to all data using nonlinear regression, and the half-maximum response (pEC_50_) of each drug, expressed as -log molar (M), was used for comparison of potency. The number of experiments is indicated by “n,” and p value < 0.05 was considered statistically significant.

## Results

### EA prevented AF-induced disorder of microvascular perfusion

**Fig. 1:**
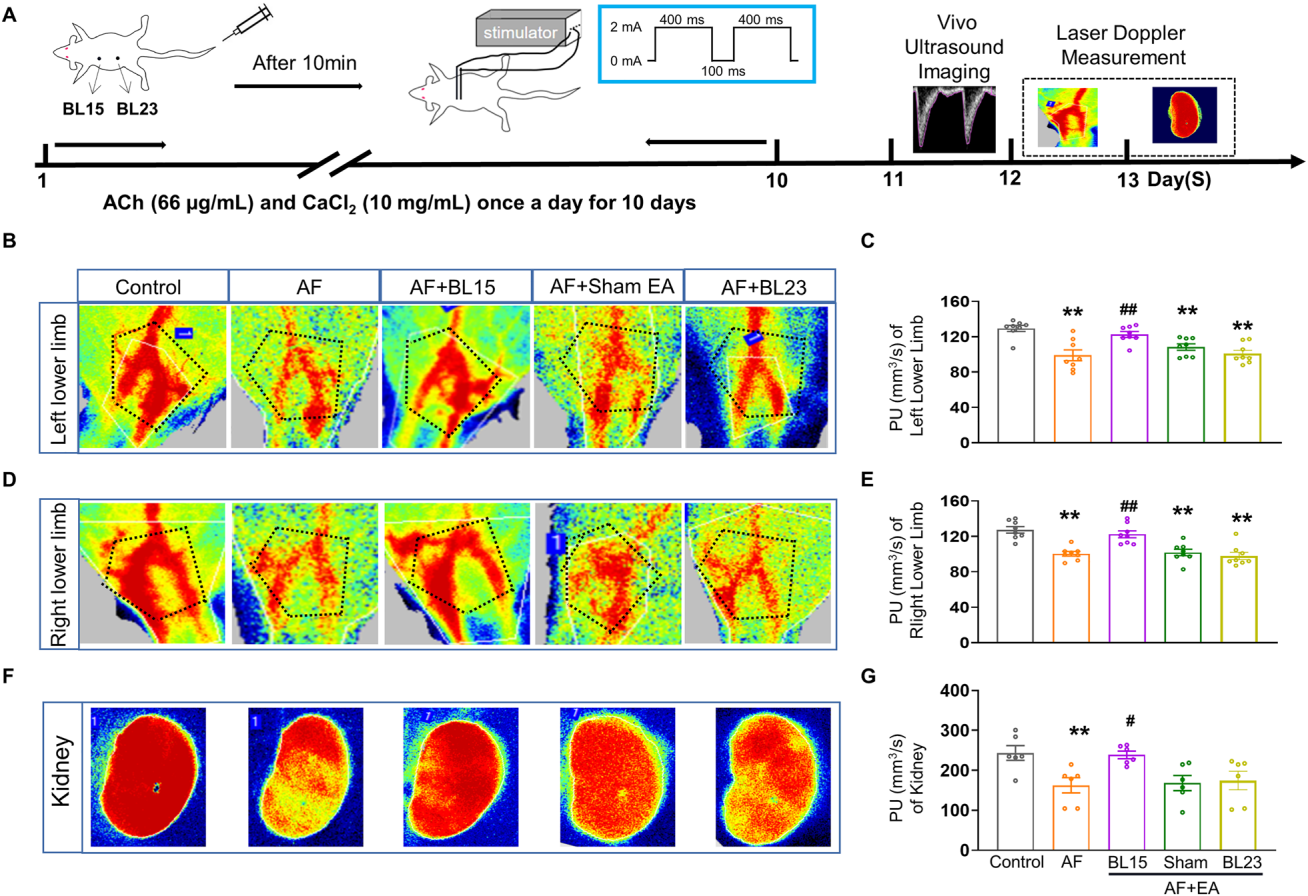
EA at BL15 improved impaired blood perfusion of lower limbs and kidneys in AF rats.** A** The experimental schedule of the AF model, EA treatment, laser Doppler measurement, and ultrasonography. **B** Representative images of blood perfusion maps in the left lower limb of each group of rats from laser speckle. **C** Quantification of the PU in the left lower limb. **D** Representative images of blood perfusion maps in the right lower limb of each group of rats from laser speckle. **E** Quantification of the PU in the right lower limb. **F** Representative images of blood perfusion maps in kidneys of each group of rats from laser speckle. **G** Quantification of the PU in kidney. BL15: Xinshu acupoint; BL23: Shenshu acupoint; EA: electroacupuncture; AF: atrial fibrillation; PU: the perfusion unit. Values are presented as means ± SEM, ^*^p < 0.05 and ^**^p < 0.01 compared with Cont., ^#^*p* < 0.05 and ^##^*p* < 0.01 compared with AF, n = 8 rats / group

To investigate the potential of EA in preventing AF-induced circulation dysfunction, we employed an AF animal model induced by ACh and CaCl_2_. We conducted ECG recordings on the isolated hearts to assess AF inducibility, duration, and effective refractory period. Rats injected with ACh-CaCl_2_ exhibited higher AF inducibility, longer AF duration, and shorter effective refractory period, indicating successful AF induction (Additional file 1: Figure S1). The BL15 is one of the back-shu points of the shaoyin heart meridian which regulates heart function [49], and Shenshu (BL23) is another back-shu acupoint which related to ‘‘kidney’’ function in traditional medicine [38]. Thus, we selected BL23 as a control point to verify the specific cardiac protective effects of BL15. Laser Doppler was used to evaluate was used to evaluate microvascular function in AF rats (Fig. 1A). The mean Perfusion Unit (PU) values of the right and left lower limbs in the AF group were significantly lower than the control group, indicative of impaired microcirculation. Remarkably, EA at BL15 prevented these AF-induced alterations in microvascular function (Fig. 1B–E). Similarly, the kidneys of AF rats exhibited decreased blood perfusion, which was also improved by EA at BL15 (Fig. 1F, G). However, sham EA and EA at BL23 did not show any improvement in PU values in the AF group (Fig. 1B–G). These results indicate that microcirculation was impaired in AF rats, and EA at BL15 effectively prevented this impairment.

### EA protected against AF-induced disorders of macrovascular perfusion

**Fig. 2:**
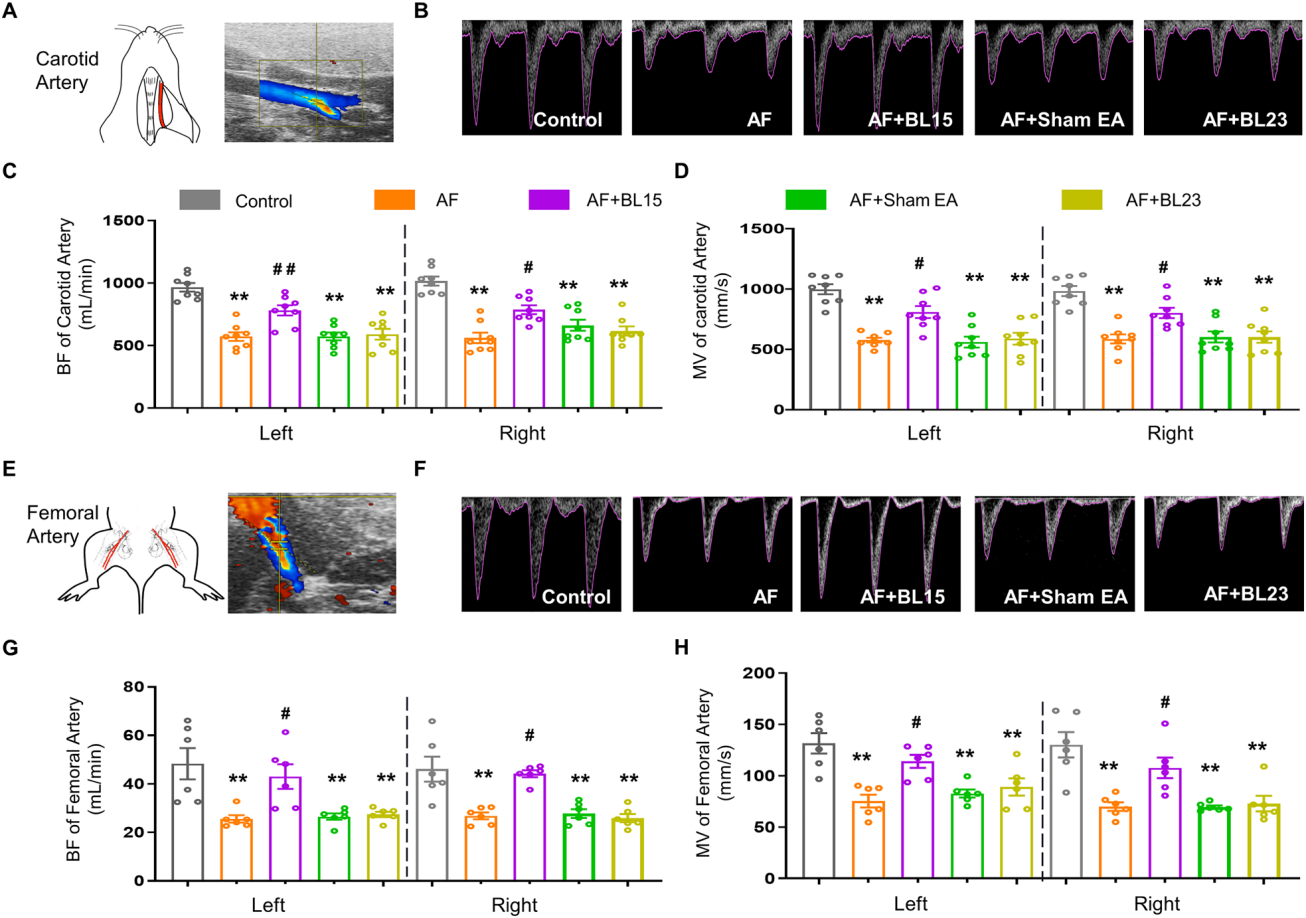
EA at BL15 ameliorated impaired blood perfusion of the carotid artery and femoral artery in AF rats.** A** Representative ultrasonography images of the femoral artery in B-Mode. **B** Representative ultrasonography images of the carotid artery in M-Mode. **C-D** Quantified values of BF **C** and MV **D** in carotid arteries of each group of rats (n = 8 rats / group). **E** Representative ultrasonography images of the femoral artery in B-Mode. **F** Representative ultrasonography images of the femoral artery in M-Mode. **G–H** Quantified values of BF **G** and MV **H** in femoral arteries of each group of rats (n = 6 rats / group). BF: blood flow; MV: mean blood velocity. Values are presented as mean ± SEM, ^*^p < 0.05 and ^**^p < 0.01 compared with Cont., ^#^*p* < 0.05 and ^##^*p* < 0.01 compared with AF

To further explore whether EA treatment could prevent the decrease in macrovascular blood perfusion induced by AF, we used ultrasonography to evaluate artery perfusion by measuring blood flow (BF) and mean velocity (MV) in the carotid and femoral arteries of rats (Fig. 2A, E). Both BF and MV values in the AF group were significantly lower than the control group in both sides of the carotid artery (Fig. 2B–D). However, EA at BL15 preserved the impaired artery perfusion. Nonetheless, sham EA and EA at BL23 did not show any improvement in the carotid artery in AF rats. Moreover, EA increased the reduced BF and MV values in femoral arteries caused by AF (Fig. 2F–H). However, there were no differences in both indexes in carotid and femoral arteries among rats from AF, AF + sham EA, and AF + EA at BL23 groups. These findings suggest that impaired macrovascular perfusion by AF can be effectively prevented by EA at BL15.

### EA restored AF-induced disability of vasomotricity in vivo

Since coagulant alterations and vascular dysfunction are major contributors to blood circulatory dysfunction, we first examined D-Dimer and fibrinogen (FG) of all experimental groups. No differences were observed in the values of D-Dimer and FG between all groups (Additional file 1: Figure S2), suggesting that ACh-CaCl_2_ administration did not affect blood coagulation in rats.

**Fig. 3:**
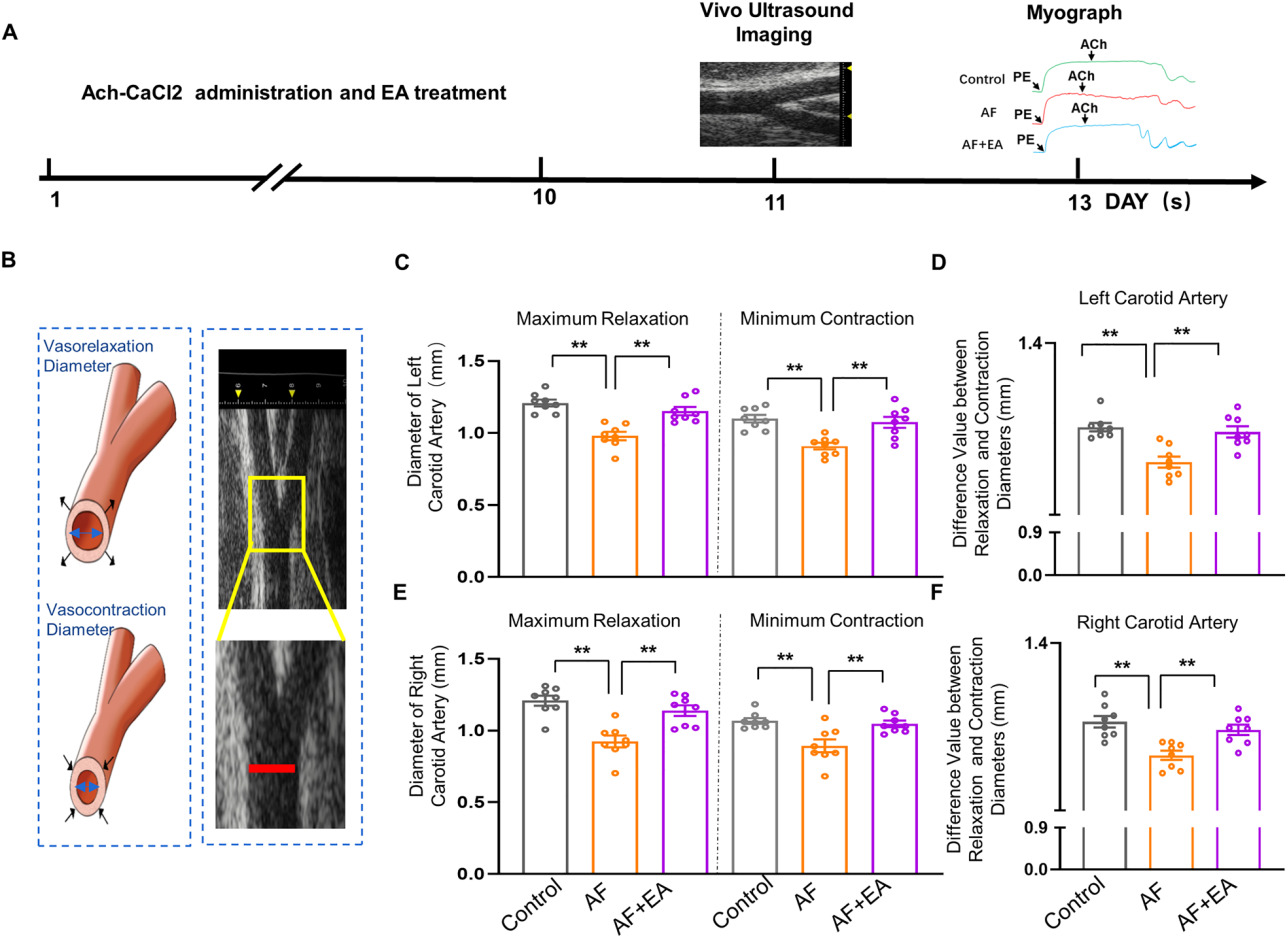
EA at BL15 prevented the reduced difference in values between relaxation and contraction diameters of the carotid artery in AF rats.** A** The experimental schedule of the AF model, EA treatment, ultrasonography, and Myograph. **B** Representative images and location of the carotid artery under in vivo ultrasound. **C** Quantified diameter values of maximum vasorelaxation and minimum contraction in the left carotid artery of each group of rats. **D** Quantification of the difference in values between relaxation and contraction diameters of the left carotid artery of each group of rats. **E** Quantified diameter values of maximum vasorelaxation and minimum contraction in the right carotid artery of each group of rats. **F** Quantification of the difference in values between relaxation and contraction diameters of right carotid artery of each group of rats. Values are presented as means ± SEM, ^*^p < 0.05 and ^**^p < 0.01, n = 8 rats / group

Next, we evaluated the dimension changes during vascular relaxation and contraction to assess vascular function [50]. We measured the minimum and maximal diameters of the carotid artery at the phase of vasoconstriction and relaxation in vivo (Fig. 3A, B). The inner diameters of vascular relaxation and contraction in the carotid artery were reduced in AF rats, and the differences between the maximum and minimum diameters of carotid arteries were decreased as well, compared with the control group (Fig. 3C–F). EA at BL15 effectively reversed these abnormal changes in AF. These data suggest that EA effectively prevented AF-induced disability of vasomotricity at BL15.

### EA did not affect AF-induced vascular endothelial dysfunction

**Fig. 4:**
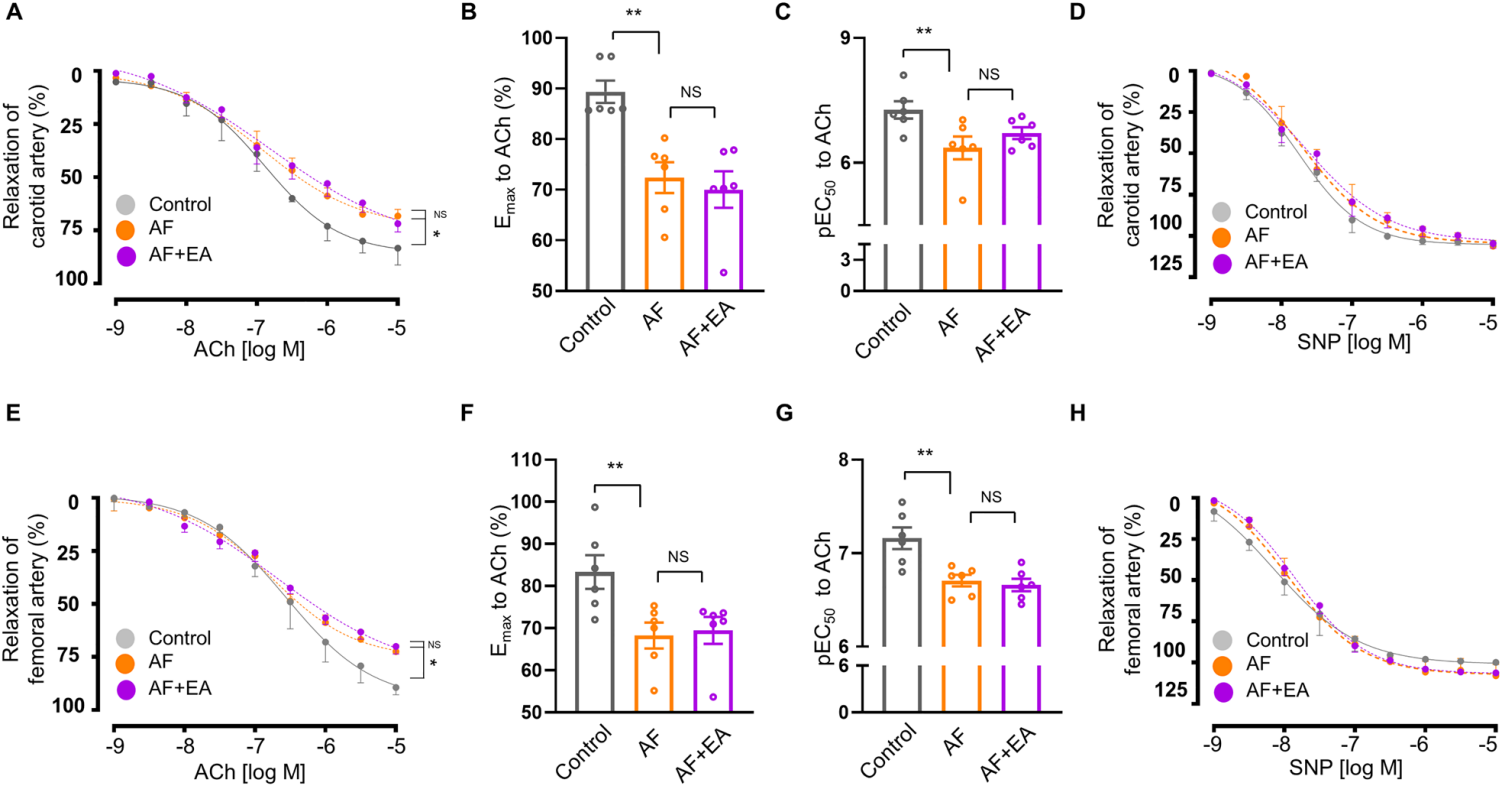
EA treatment had no effect on vascular endothelial dysfunction in AF rats. **A** Endothelium-dependent vasorelaxation curve of ACh. **B** Quantification of ACh E_max_ in the carotid artery of each group of rats. **C** Quantification of ACh pEC_50_. **D** Endothelium-independent vasorelaxation curve of SNP in carotid arteries of each group of rats. **E** Endothelium-dependent vasorelaxation curve of ACh. **F** Quantification of ACh E_max_. **G** Quantification of ACh pEC_50_. **H** Endothelium-independent vasorelaxation curve of SNP in femoral artery of each group of rats. ACh: acetylcholine, used to study endothelial-dependent vasodilators; SNP: sodium nitroprusside, a nitric oxide donor, used to study endothelial-independent vasodilators; E_max_: the value of maximal relaxation; pEC_50_: negative logarithm of EC_50_. Values are presented as means ± SEM, ^*^p < 0.05 and ^**^p < 0.01, n = 6 rats / group

The function of both endothelial and smooth muscle cells are key factors in regulating vascular relaxation and contraction function. To delve into the underlying mechanism of EA's improved vascular dysfunction, we first analyzed vascular endothelial function using an endothelial-dependent vasodilator (ACh) and non-endothelial function using sodium nitroprusside (SNP, NO donor) in isolated carotid and femoral arteries of rats from all groups. We found a significant endothelial vascular dysfunction in the carotid arteries of AF rats, indicated by a decrease in maximal relaxation (E_max_) and pEC_50_ values in response to ACh. Surprisingly, EA at BL15 did not restore these deficits of endothelial-dependent vasorelaxation in AF rats (Fig. 4A-C). However, there were no differences in the SNP curves among all groups (Fig. 4D), suggesting that EA at BL15 may not affect AF-induced vascular endothelial dysfunction.

Similar results were observed in the femoral artery, where rats from both AF and AF + EA groups exhibited vascular endothelial dysfunction, as evidenced by lower E_max_ and higher EC_50_ values in response to ACh, compared to the control group (Fig. 4E–G). Furthermore, rats from all groups showed similar relaxation curves in response to SNP (Fig. 4H). Together, these results suggest that neither endothelial cells nor smooth muscle cell function seems to be involved in the protective effect of EA against circulatory injury.

### EA prevented AF-induced abnormal activation of the autonomic nervous system

**Fig. 5:**
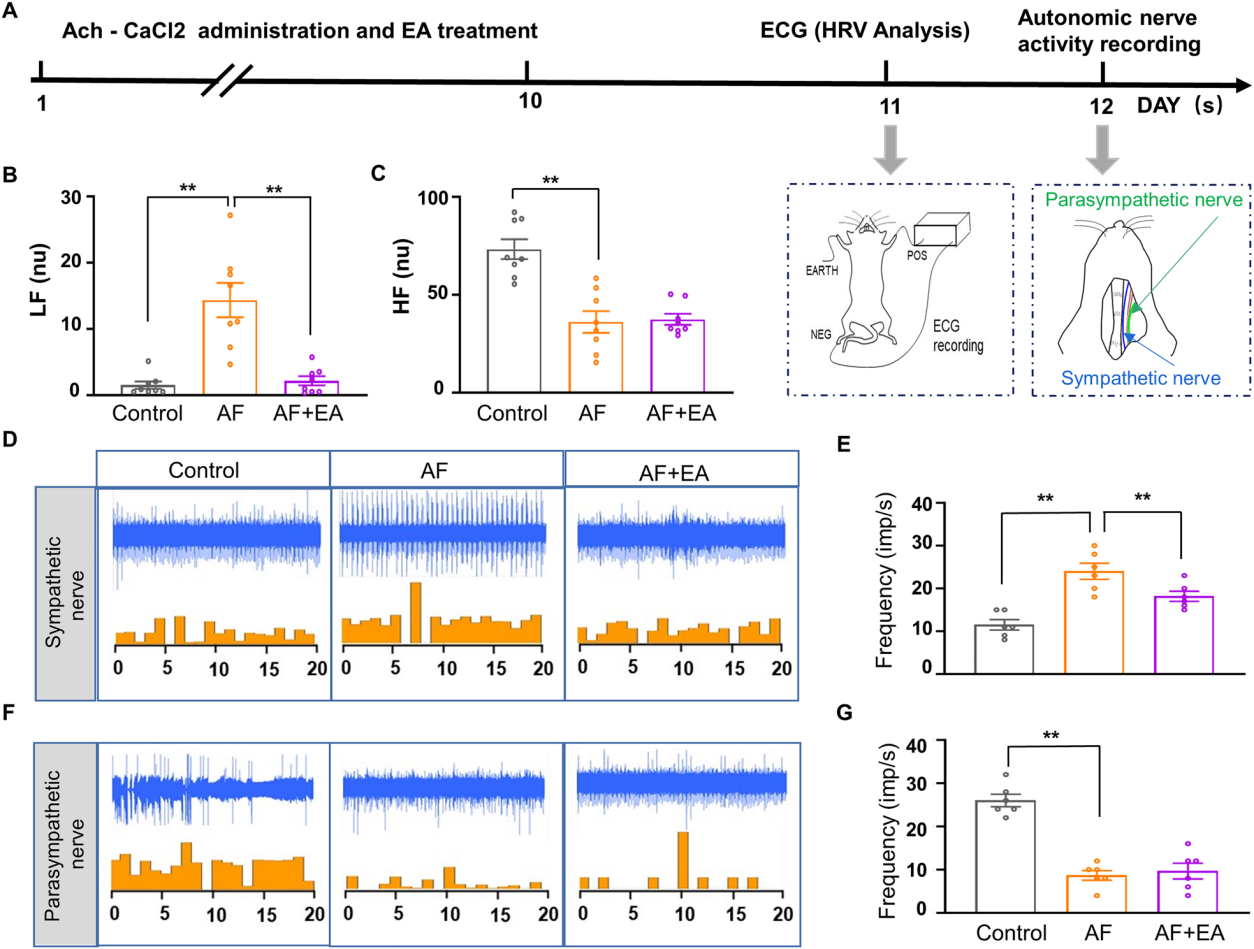
EA treatment altered abnormal autonomic nerve activity in AF rats. **A** The experimental schedule of HRV analysis and autonomic nerve activity recording in vivo. **B–C** Quantification of LF **B** and HF **C** in each group. **D** Representative activity of cervical sympathetic nerve in each group of rats. **E** Quantification of sympathetic nerve spike frequency. **F** Representative activity of cervical vagus nerve in each group of rats. **G** Quantification of vagus nerve spike frequency. HRV: Heart rate variability; LF: low frequency, HF: high frequency; LF:low frequency in normalized units; HF: high frequency in normalized units. Values are presented as means ± SEM, ^*^p < 0.05 and ^**^p < 0.01, n = 6 rats/group

Autonomic nervous regulation plays a crucial role in vascular relaxation and contraction [51]. To investigate the involvement of the autonomic nervous system in EA treatment, we analyzed heart rate variability (HRV) using electrocardiogram (ECG) (Fig. 5A). AF rats exhibited a higher percentage of low-frequency (LF) and a lower percentage of high-frequency (HF) components in total frequency compared to the control group (Fig. 5B, C). Notably, AF + EA rats showed a decreased percentage of LF compared to AF rats, but there was no difference in the percentage of HF between the two groups. These findings suggest that EA treatment may modulate autonomic nervous activity.

To further elucidate the role of autonomic nerve activity in EA treatment, we recorded the activity of the cervical sympathetic and vagus nerve in vivo (Fig. 5A). In AF rats, the spike frequency of the sympathetic nerve was significantly increased, while the spike frequency of the vagus nerve was decreased compared to the control group (Fig. 5D–G). Notably, EA at BL15 significantly reduced the sympathetic nerve spike frequency in AF rats, but it had no effect on vagal activity. These results indicate that EA treatment can modify the abnormal sympathetic activity caused by AF, but it does not affect the activity of the vagus nerve.

### Excessive PVN Neuron activation blocked the protective effects of EA on circulation dysfunction

**Fig. 6:**
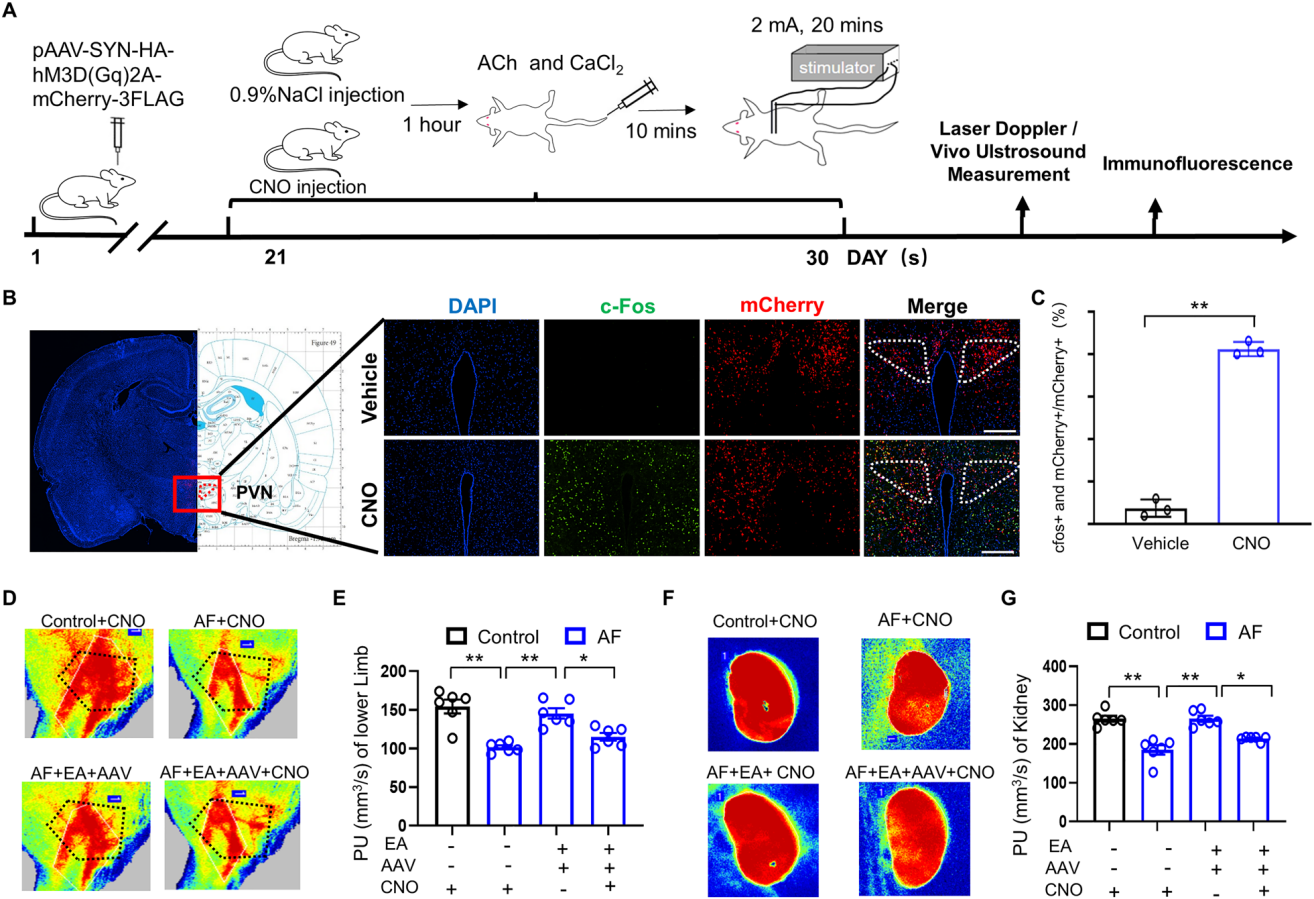
Excessive sympathetic activation neutralized the protection of EA in microcirculation dysfunction. **A** The experimental schedule of the validation test whereby pAAV-SYN-HA-hM3D(Gq)2A-mCherry-3FLAG was injected, followed by ultrasonography, laser doppler measurement in vivo, and the immunofluorescence test. **B** Representative images of c-Fos positive cells (green) and mCherry positive cells (red) in the PVN in each group of rats (Scale bar: 200 μm). **C** Quantification of positive-staining cells (n = 3 rats/group). **D** Representative images of blood perfusion maps in the left lower limb of each group of rats by laser speckle. **E** Quantification of blood perfusion in the left lower limb (n = 6 rats/group). **F** Representative images of blood perfusion maps in the kidneys of each group of rats by laser speckle. **G** Quantification of PU in kidneys (n = 6 rats/group). PVN: paraventricular nucleus of hypothalamus. Values are presented as means ± SEM, ^*^p < 0.05 and ^**^p < 0.01

To explore the role of PVN activation in the protection against AF-induced microcirculation dysfunction by EA treatment, we bilaterally injected pAAV-SYN-HA-hM3D(Gq)2A-mCherry-3FLAG (hM3D(Gq)2A) into the PVN to activate neurons with CNO (Fig. 6A). The number of c-Fos immunoreactive neurons in the PVN was significantly increased, confirming the excessive activation of neurons in this region (Fig. 6B, C). We then investigated whether elevated sympathetic activity could block the effects of EA treatment. AF rats that received CNO treatment exhibited lower PU values in the lower limbs compared to control rats (Fig. 6D, E). Interestingly, EA significantly improved blood perfusion in the lower limbs of AF rats with hM3D(Gq)2A virus injection into the PVN. Importantly, the administration of CNO or hM3D(Gq)2A virus alone did not alter the effect of ACh-CaCl_2_ or EA treatment on PU. However, the combination of CNO and AAV inhibited the improvement of PU in the lower limbs of AF rats treated with EA. Similar results were observed in the kidneys (Fig. 6F, G).

**Fig. 7:**
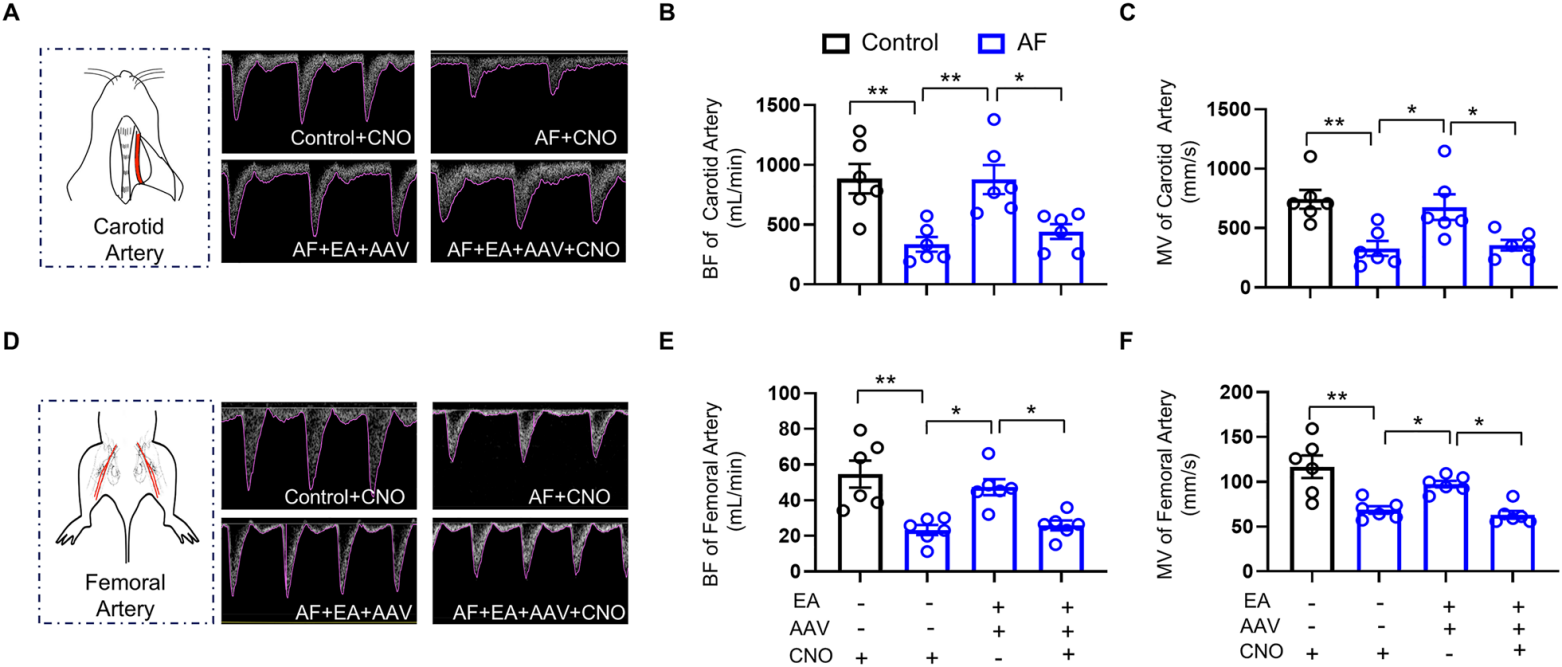
Excessive sympathetic activation neutralized the protection of EA in macrocirculation dysfunction. **A** Representative ultrasonography images of the carotid artery in M-Mode. **B-C** Quantification of BF **B** and MV **C** in carotid arteries of each group of rats. **D** Representative ultrasonography images of the femoral artery in M-Mode. **E–F** Quantification of BF **E** and MV **F** in femoral arteries of each group of rats. BF: blood flow; MV: mean blood velocity. Values are presented as means ± SEM, ^*^p < 0.05 and ^**^p < 0.01, n = 6 rats/group

To further validate whether the improvement of EA on AF-induced macrocirculatory dysfunction is mediated by PVN activation, we performed ultrasonography on carotid and femoral arteries of rats. Consistent with the microcirculation results, remarkable decreases in BF and MV values were observed in the carotid arteries of AF rats with CNO administration. EA treatment increased BF and MV values in AF rats with hM3D(Gq)2A virus injection alone (Fig. 7A–C). The injection of hM3D(Gq)2A virus combined with CNO blocked the protective effects of EA in the femoral artery (Fig. 7A–C). Similar results were observed in BF and MV values of the femoral artery (Fig. 7D–F). These findings collectively suggest that modulating the activity of PVN neurons is crucial in preventing AF-induced circulation dysfunction by EA at BL15.

## Discussion 

In this study, we have provided evidence of macro- and microvascular dysfunction in ACh-CaCl_2_ induced AF rats. This is consistent with AF patients who experience an increased risk of pathological thrombus formation due to circulatory disturbances, including abnormal blood constituents, vessel wall lesions, and reduced blood flow [6, 52]. Recent clinical research has shown that reduced blood flow is more significant in aged patients with AF [53]. Additionally, AF experimental models, such as CREM-TG mice, have shown vascular inflammation and impaired pulmonary circulation [54]. Our study demonstrates that EA treatment at BL15 effectively improves both macro- and microvascular function in this AF rat model. Acupuncture treatment has also been reported to improve impaired circulation in patients with heart failure, reduce experimental renovascular hypertension by ameliorating endothelial cell injury, and increase blood perfusion in cerebral blood vessels and heart under ischemic conditions [55–57]. Moreover, acupuncture or EA at BL15 has been shown to effectively treat vascular injury in patients or animal models with various cardiovascular diseases, including chronic heart failure, hypertension, and myocardial ischemia [34, 58, 59].

Although both blood viscosity and vascular function are closely related to blood circulation in AF [1, 60], our study found no changes in D-Dimer and FG levels in the plasma of rats from all groups, suggesting that our modeling protocol did not cause abnormal changes in blood constituents. We speculate that the different results reported in the literature and our study may be related to variations in age, sex, or pathological status [60, 61]. For example, clinical studies have shown that AF patients with changes in FG and D-dimer levels usually have other comorbidities, such as diabetes mellitus and hypertension [60, 62]. However, our results support that EA treatment can reverse the disability of vasomotricity caused by AF in this study. Furthermore, we found that AF rats presented deficits of endothelial-dependent vasorelaxation compared to controls. Endothelial function is well known to be a vital factor in maintaining vascular function [63], and the presence of AF has been independently correlated with impaired vascular endothelial function in clinical settings [64]. Surprisingly, AF-induced vascular endothelial dysfunction could not be ameliorated by EA treatment. These results demonstrate that the mechanism of EA protection against blood flow dysfunction may be related to the modulation of vessel function rather than endothelial impairment.

In line with our previous study, we found enhanced sympathetic activity and decreased vagus nerve activity in rats with ACh-CaCl_2_ administration (Figs. 2, 3). This is consistent with previous findings in which subcutaneous nerve stimulation remodeled the stellate ganglion and reduced stellate ganglion nerve activity in dogs with acute myocardial infarction to regulate cardiac activity. In clinical settings, transcutaneous electrical nerve stimulation (TENS) attenuates blood pressure and vasoconstrictor responses in healthy young and older individuals, and HRV analysis showed an improvement in sympatho-vagal balance [65]. Low-frequency TENS decreases sympathetic nervous system activity and increases parasympathetic nervous system activity when applied to the paravertebral ganglionar region in hypertensive patients [66]^.^ Additionally, cervicothoracic transcutaneous electrical nerve stimulation attenuates the cardiac sympathetic overdrive in patients with heart failure [67]^.^ Collectively, these results indicate that the abnormal activity of the sympathetic nervous system (SNS) is responsible for AF and AF-induced circulatory dysfunction.

Recent studies have demonstrated that acupuncture or EA treatment can modulate autonomic nerve activity, including heart rate, heart rate variability, blood pressure, cardiac function, infarct size of the heart, and pupil size [55, 68–72]. Furthermore, EA suppresses cardiac excitability and modulates the imbalance of autonomic tone induced by left stellate ganglion stimulation (LSS) in a porcine model [73]. Previous studies have reported that EA stimulated at BL15 (2 Hz) significantly increased the normalized high-frequency power component of HRV, while decreasing the low-frequency power in healthy volunteers [74], indicating that the imbalance between parasympathetic and sympathetic activity may be modulated. However, we found that EA treatment at BL15 downregulated the enhancement of sympathetic activity, as indicated by a higher percentage of LF and increased spike frequency of the sympathetic nerve, but did not alter the reduced vagus nerve activity caused by ACh-CaCl_2_ administration. Moreover, another study reported that manual acupuncture and electromagnetic field stimulation at BL15 activated the parasympathetic nervous system, while laser acupuncture at BL15 activated the sympathetic nervous system [49]. These findings suggest that EA at BL15 has different regulatory effects on the autonomic nervous system and may be attributable to variations in species, pathological states, and stimulation modes.

Despite being considered the ‘‘autonomic master controller,’’ the role of the PVN in the modulation of sympathetic nervous activity by BL15 stimulation has remained unclear. Our results provide evidence for the first time that PVN can be regulated by BL15 stimulation, which affects the control of blood circulation through the sympathetic system. Previous studies have also reported that PVN inhibition can block the alteration of the values of LF and HF during the pulse interval in response to acute microgravity (head-down tilt) in the same study [75]. Moreover, pharmacological or chemical stimulation of the PVN results in rapid increases in heart rate, blood pressure, or renal sympathetic nerve activity [76–78]. Our current study demonstrates that the protective effect of EA on AF-induced circulatory dysfunction is related to inhibiting enhanced PVN activity. Inconsistent with our data, EA treatment has been shown to decrease barosensitive PVN neurons and increase blood pressure [79], and EA can alleviate myocardial injury through the PVN nerve pathway [37, 80]. PVN processes have also been shown to participate in EA-modulation of neural-mediated vasodepression and bradycardia [81]. EA has been reported to promote cardiac function and decrease PVN neuron activity in rats with surgery-induced stress [82]. Our study provides evidence that EA can regulate PVN to treat cardiovascular and circulatory diseases. Ultimately, these findings are consistent with the purpose of the United States Common Fund's Stimulating Peripheral Activity to Relieve Conditions program [83], which aims to use peripheral stimulation to treat peripheral and central diseases.

## Conclusions

In conclusion, our study demonstrates that AF-induced circulatory dysfunction is effectively protected by EA at BL15. EA treatment also restores the disability of vasomotricity and elevated sympathetic activity in AF rats. Additionally, excessive sympathetic activation by chemogenetic manipulation of PVN neurons blocked the protective effects of EA treatment. In summary, our results indicate that the effect of EA at BL15 in preventing circulatory dysfunction is related to the modulation of PVN neuron activity.

### Supplementary Information

**Additional file 1:** Effect of ACh-CaCl_2_ on rats. **Figure S1.** Atrial fibrillation has been successfully induced by ACh-CaCl_2_ treatment in rats. **Figure S2.** ACh-CaCl_2_ administration did not affect blood coagulation in rats.

## Data Availability

The datasets used or analyzed throughout this study are available from the corresponding author upon reasonable request.

## Abbreviations

EA: Electroacupuncture
FA: Fibrillation
PVN: Paraventricular nucleus
BL15: Xinshu
BL23: Shenshu
Ach: Acetylcholine
ECG: Electrocardiogram
ERP: Effective refractory period
MV: Mean blood velocity
RFA: Right femoral artery
LFA: Left femoral artery
ROI: Regions of interest
PU: Perfusion unit
AAV: Adeno-associated virus
LF: Low frequency

## References

[1] Khan AA (2020). Endothelial function in patients with atrial fibrillation. Ann Med.

[2] Ovsenik A, Podbregar M, Fabjan A (2021). Cerebral blood flow impairment and cognitive decline in heart failure. Brain Behav.

[3] Watson T, Shantsila E, Lip GY (2009). Mechanisms of thrombogenesis in atrial fibrillation: Virchow's triad revisited. Lancet.

[4] Shariff N (2012). AF and Venous Thromboembolism—pathophysiology, risk assessment and CHADS-VASc score. J Atr Fibrillation.

[5] Tsai LM, Chen JH, Tsao CJ (1998). Relation of left atrial spontaneous echo contrast with prethrombotic state in atrial fibrillation associated with systemic hypertension, idiopathic dilated cardiomyopathy, or no identifiable cause (lone). Am J Cardiol.

[6] Brotman DJ (2004). Virchow's triad revisited. South Med J.

[7] Nozawa T (2006). D-dimer level influences thromboembolic events in patients with atrial fibrillation. Int J Cardiol.

[8] Freestone B (2007). Soluble E-selectin, von Willebrand factor, soluble thrombomodulin, and total body nitrate/nitrite product as indices of endothelial damage/dysfunction in paroxysmal, persistent, and permanent atrial fibrillation. Chest.

[9] Krishnamoorthy S (2013). Prognostic role of plasma von Willebrand factor and soluble E-selectin levels for future cardiovascular events in a 'real-world' community cohort of patients with atrial fibrillation. Eur J Clin Invest.

[10] You JJ (2012). Antithrombotic therapy for atrial fibrillation: antithrombotic therapy and prevention of thrombosis, 9th ed: American college of chest physicians evidence-based clinical practice guidelines. Chest.

[11] Moss JD, Cifu AS (2015). Management of Anticoagulation in Patients With Atrial Fibrillation. JAMA.

[12] Urena M (2013). Percutaneous left atrial appendage closure with the AMPLATZER cardiac plug device in patients with nonvalvular atrial fibrillation and contraindications to anticoagulation therapy. J Am Coll Cardiol.

[13] Saw J (2019). Antithrombotic therapy and device-related thrombosis following endovascular left atrial appendage closure. JACC Cardiovasc Interv.

[14] Ferguson AV, Latchford KJ, Samson WK (2008). The paraventricular nucleus of the hypothalamus—a potential target for integrative treatment of autonomic dysfunction. Expert Opin Ther Targets.

[15] Coote JH (2005). A role for the paraventricular nucleus of the hypothalamus in the autonomic control of heart and kidney. Exp Physiol.

[16] Swanson LW, Sawchenko PE (1980). Paraventricular nucleus: a site for the integration of neuroendocrine and autonomic mechanisms. Neuroendocrinology.

[17] Dampney RA (2005). Long-term regulation of arterial blood pressure by hypothalamic nuclei: some critical questions. Clin Exp Pharmacol Physiol.

[18] Savic B, Murphy D, Japundzic-Zigon N (2022). The paraventricular nucleus of the hypothalamus in control of blood pressure and blood pressure variability. Front Physiol.

[19] Jia S (2015). Involvement of the paraventricular nucleus in the occurrence of arrhythmias in middle cerebral artery occlusion rats. J Stroke Cerebrovasc Dis.

[20] Kc P, Dick TE (2010). Modulation of cardiorespiratory function mediated by the paraventricular nucleus. Respir Physiol Neurobiol.

[21] Mancia G, Grassi G (2014). The autonomic nervous system and hypertension. Circ Res.

[22] Shen MJ, Zipes DP (2014). Role of the autonomic nervous system in modulating cardiac arrhythmias. Circ Res.

[23] Xu B, Zheng H, Patel KP (2012). Enhanced activation of RVLM-projecting PVN neurons in rats with chronic heart failure. Am J Physiol Heart Circ Physiol.

[24] Yang Su (2022). Restoring the autonomic balance in an atrial fibrillation rat model by electroacupuncture at the neiguan point. Neuromodulation.

[25] Chen M (2015). Noninvasive vagus nerve stimulation: a novel promising modulator for cardiac autonomic nerve system dysfunction. Int J Cardiol.

[26] Ardell JL (2017). Defining the neural fulcrum for chronic vagus nerve stimulation: implications for integrated cardiac control. J Physiol.

[27] Bang SK (2018). Attenuation of hypertension by C-fiber stimulation of the human median nerve and the concept-based novel device. Sci Rep.

[28] De Ferrari GM (2011). Chronic vagus nerve stimulation: a new and promising therapeutic approach for chronic heart failure. Eur Heart J.

[29] Cabioglu MT, Arslan G (2008). Neurophysiologic basis of Back-Shu and Huatuo-Jiaji points. Am J Chin Med.

[30] Ni YM, Frishman WH (2018). Acupuncture and cardiovascular disease: focus on heart failure. Cardiol Rev.

[31] Liu Y (2019). Acupuncture therapy for the treatment of stable angina pectoris: an updated meta-analysis of randomized controlled trials. Complement Ther Clin Pract.

[32] Lee JM (2021). Effects of acupuncture on cardiac remodeling in patients with persistent atrial fibrillation: results of a randomized, placebo-controlled, patient- and assessor-blinded pilot trial and its implications for future research. Medicina.

[33] Guo X (2022). Modulation of brain rhythm oscillations by xingnao kaiqiao acupuncture correlates with stroke recovery: a randomized control trial. J Integr Complement Med..

[34] Li M (2012). Effects of electroacupuncture at PC6 and BL15 on nerve electrical activity in spinal dorsal root and norepinephrine and dopamine contents in paraventricular nucleus of hypothalamus in rats with acute myocardial ischemia. Zhong Xi Yi Jie He Xue Bao.

[35] Xu L (2013). Treating angina pectoris by acupuncture therapy. Acupunct Electrother Res.

[36] Gao Z (2013). Treating coronary heart disease by acupuncture at neiguan (PC6) and xinahu (BL15): an efficacy assessment by SPECT. Zhongguo Zhong Xi Yi Jie He Za Zhi.

[37] Cui S (2018). Electroacupuncture improved the function of myocardial ischemia involved in the hippocampus-paraventricular nucleus-sympathetic nerve pathway. Evid Based Complement Alternat Med.

[38] Zhang Z (2020). Correlated sensory and sympathetic innervation Between the acupoint BL23 and kidney in the rat. Front Integr Neurosci.

[39] Wang J (2021). Protection against doxorubicin-induced cardiotoxicity through modulating iNOS/ARG 2 balance by electroacupuncture at PC6. Oxid Med Cell Longev.

[40] Chan YH (2019). Atrial fibrillation and its arrhythmogenesis associated with insulin resistance. Cardiovasc Diabetol.

[41] Hiram R (2019). Right atrial mechanisms of atrial fibrillation in a rat model of right heart disease. J Am Coll Cardiol.

[42] Ding Y (2019). Peroxynitrite-mediated SIRT (Sirtuin)-1 inactivation contributes to nicotine-induced arterial stiffness in Mice. Arterioscler Thromb Vasc Biol.

[43] Trojnar E (2020). Cannabinoid-2 receptor activation ameliorates hepatorenal syndrome. Free Radic Biol Med.

[44] Cheng H (2020). HuangqiGuizhiWuwu decoction prevents vascular dysfunction in diabetes via inhibition of endothelial arginase 1. Front Physiol.

[45] Spronk HM (2017). Hypercoagulability causes atrial fibrosis and promotes atrial fibrillation. Eur Heart J.

[46] Billman GE (2011). Heart rate variability—a historical perspective. Front Physiol.

[47] Stauss HM (2006). Baroreceptor reflex sensitivity estimated by the sequence technique is reliable in rats. Am J Physiol Heart Circ Physiol.

[48] Cui X (2022). Referred somatic hyperalgesia mediates cardiac regulation by the activation of sympathetic nerves in a rat model of myocardial ischemia. Neurosci Bull.

[49] Lee NR (2016). Comparison of the effects of manual acupuncture, laser acupuncture, and electromagnetic field stimulation at acupuncture point BL15 on heart rate variability. J Acupunct Meridian Stud.

[50] Li M (2018). Endothelial-vascular smooth muscle cells interactions in atherosclerosis. Front Cardiovasc Med.

[51] Amiya E, Watanabe M, Komuro I (2014). The relationship between vascular function and the autonomic nervous system. Ann Vasc Dis.

[52] Kochiadakis GE, Kallergis EM (2012). Impact of atrial fibrillation on coronary blood flow: a systematic review. J Atr Fibrillation.

[53] Deyranlou A (2021). Effects of ageing on aortic circulation during atrial fibrillation; a numerical study on different aortic morphologies. Ann Biomed Eng.

[54] Bukowska A (2018). CREM-transgene mice: an animal model of atrial fibrillation and thrombogenesis. Thromb Res.

[55] Lee H, Kim TH, Leem J (2016). Acupuncture for heart failure: a systematic review of clinical studies. Int J Cardiol.

[56] Kim DD (2006). Acupuncture reduces experimental renovascular hypertension through mechanisms involving nitric oxide synthases. Microcirculation.

[57] Zhuang Y (2022). Influence of acupuncture on microcirculation perfusion of pericardium meridian and heart in acute myocardial ischemia model rats. Chin J Integr Med.

[58] Wei W (2022). Effects of moxibustion at bilateral Feishu (BL13) and Xinshu (BL15) combined with benazepril on myocardial cells apoptosis index and apoptosis-related proteins cytochrome c and apoptosis-inducing factor in rats with chronic heart failure. J Tradit Chin Med.

[59] Research on the impact of acupuncture on vascular endothelium through the Rhoa/ROCK signal pathway in spontaneously hyperetensive rats (2018). Guangzhou.

[60] Weymann A (2017). Predictive role of coagulation, fibrinolytic, and endothelial markers in patients with atrial fibrillation, stroke, and thromboembolism: a meta-analysis, meta-regression, and systematic review. Med Sci Monit Basic Res.

[61] Akoum N (2018). Age and sex differences in atrial fibrosis among patients with atrial fibrillation. Europace.

[62] Jame S, Barnes G (2020). Stroke and thromboembolism prevention in atrial fibrillation. Heart.

[63] Galley HF, Webster NR (2004). Physiology of the endothelium. Br J Anaesth.

[64] Kanazawa H (2021). Improvement of vascular endothelial function reflects nonrecurrence after catheter ablation for atrial fibrillation. J Am Heart Assoc.

[65] Vieira PJC (2012). Effect of transcutaneous electrical nerve stimulation on muscle metaboreflex in healthy young and older subjects. Eur J Appl Physiol.

[66] Do ASS (2018). Effects of transcutaneous electrical nerve stimulation in autonomic nervous system of hypertensive patients: a randomized controlled trial. Curr Hypertens Rev.

[67] Campos MO (2019). Transcutaneous electrical nerve stimulation attenuates cardiac sympathetic drive in heart failure: a (123)MIBG myocardial scintigraphy randomized controlled trial. Am J Physiol Heart Circ Physiol.

[68] Hsieh CL (1999). Changes of pulse rate and skin temperature evoked by electroacupuncture stimulation with different frequency on both Zusanli acupoints in humans. Am J Chin Med.

[69] Jones AY (2011). Electrical stimulation of acupuncture points and blood pressure responses to postural changes: a pilot study. Am J Crit Care.

[70] Tachibana K (2012). Randomized comparison of the therapeutic effect of acupuncture, massage, and tachibana-style-method on stiff shoulders by measuring muscle firmness, VAS, pulse, and blood pressure. Evid Based Complement Alternat Med.

[71] Ohsawa H (1997). Neural mechanism of pupillary dilation elicited by electro-acupuncture stimulation in anesthetized rats. J Auton Nerv Syst.

[72] Lu SF (2021). Electroacupuncture improves cardiac function and reduces infarct size by modulating cardiac autonomic remodeling in a mouse model of myocardial ischemia. Acupunct Med.

[73] Takamiya T (2018). Effect of electroacupuncture on porcine cardiac excitability induced by left stellate ganglion stimulation. Auton Neurosci.

[74] Hsu CC (2006). Effects of electrical acupuncture on acupoint BL15 evaluated in terms of heart rate variability, pulse rate variability and skin conductance response. Am J Chin Med.

[75] Amorim ED (2015). Functional evidence of paraventricular nucleus involvement in cardiovascular and autonomic modulation in response to acute microgravity (head-down tilt) in unanesthetized rats. J Neurosci Res.

[76] Martin DS, Haywood JR (1993). Hemodynamic responses to paraventricular nucleus disinhibition with bicuculline in conscious rats. Am J Physiol.

[77] Zhang K, Mayhan WG, Patel KP (1997). Nitric oxide within the paraventricular nucleus mediates changes in renal sympathetic nerve activity. Am J Physiol.

[78] Kawabe T (2009). Mechanism of heart rate responses elicited by chemical stimulation of the hypothalamic paraventricular nucleus in the rat. Brain Res.

[79] Tjen-A-Looi SC (2016). Paraventricular nucleus modulates excitatory cardiovascular reflexes during electroacupuncture. Sci Rep.

[80] Xiaotong W (2022). Electroacupuncture preconditioning alleviates myocardial ischemia-reperfusion injury through the hypothalamic paraventricular nucleus- interposed nucleus nerve pathway. J Tradit Chin Med.

[81] Tjen-A-Looi SC (2022). Neurogenic hypotension and bradycardia modulated by electroacupuncture in hypothalamic paraventricular nucleus. Front Neurosci.

[82] Zhang HH (2012). Protective effects of electroacupuncture on cardiac function in rats subjected to thoracic surgery trauma. Brain Res Bull.

[83] Stimulating peripheral activity to relieve conditions (SPARC). 2022. 10.3389/fninf.2022.819198 PMC9449460 36090663

