# Supplementary material for: Modulating activity of PVN neurons prevents atrial fibrillation induced circulation dysfunction by electroacupuncture at BL15
Source: Chin Med. 2023 Oct 17;18:135. doi: 10.1186/s13020-023-00841-6 (PMC10580609; doi:10.1186/s13020-023-00841-6)
Supplement: Supplementary file 1 — Additional file 1: Effect of ACh-CaCl2 on rats. Figure S1. Atrial fibrillation has been successfully induced by ACh-CaCl2 treatment in rats. Figure S2. ACh-CaCl2 administration did not affect blood coagulation in rats. [file 13020_2023_841_MOESM1_ESM.docx]

## Additional file Materials: Effect of ACh-CaCl_2_ on rats.


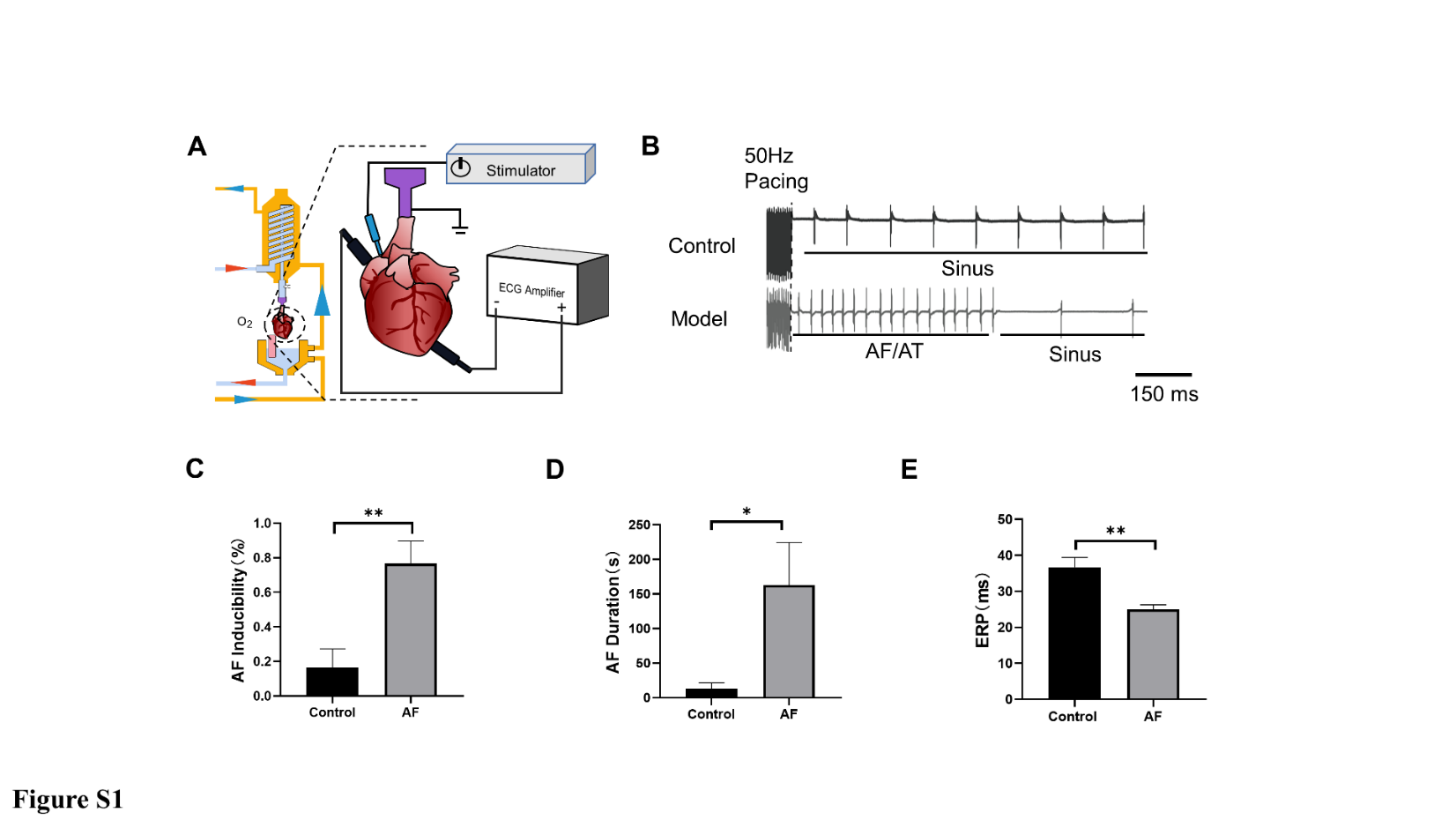


**Figure S1. Atrial fibrillation has been successfully induced by ACh-CaCl_2_ treatment in rats. A** ECG detection in an isolated rat's heart. **B** Atrial fibrillation induced by 50 Hz electrical stimulation. **C** Incidence of atrial fibrillation. **D** Duration of atrial fibrillation. E Effective refractory period. ECG: electrocardiogram. Values are presented as means ± SEM, ^*^*p*<0.05 and ^**^*p*<0.01, n=6 rats / group.


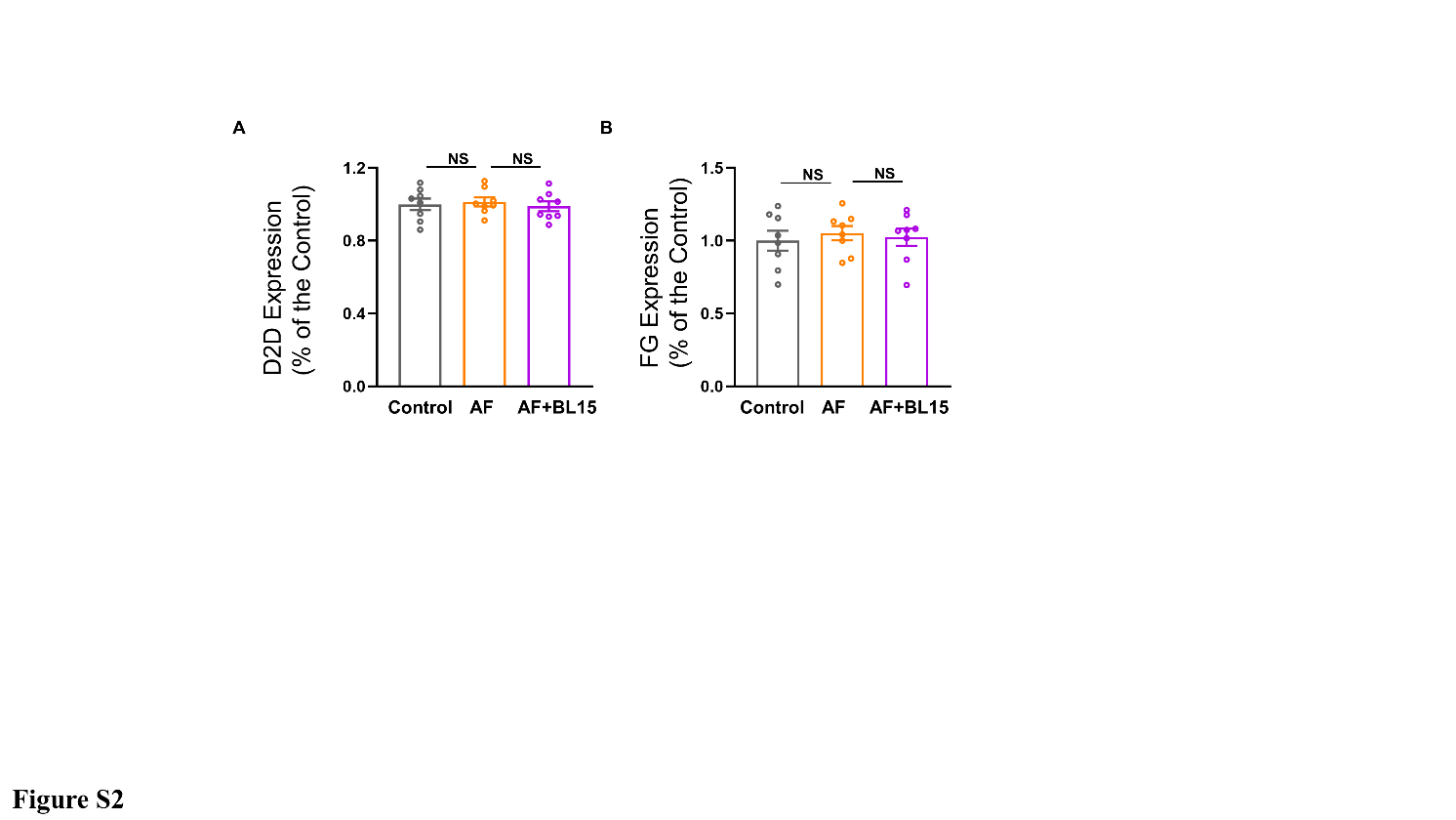


**Figure S2. ACh-CaCl_2_ administration did not affect blood coagulation in rats. A-B** The results of the statistical analysis for the contents of D2D (A) and FG (B). D2D: D-Dimer; FG: fibrinogen; Values are presented as means ± SEM, ^*^*p*<0.05 and ^**^*p*<0.01, n=8 rats / group.
